# Long non-coding RNA GNAS-AS1 knockdown inhibits proliferation and epithelial–mesenchymal transition of lung adenocarcinoma cells via the microRNA-433-3p/Rab3A axis

**DOI:** 10.1515/med-2023-0740

**Published:** 2023-07-14

**Authors:** Jing He, Xiaoxiang Xi, Peng Cao, Jinxia Zhou, Hui Liu, Na Li

**Affiliations:** Department of Thoracic Surgery, Taixing People’s Hospital, Taixing, 225400, China; Department of Thoracic Surgery, Taixing People’s Hospital, No. 1 Changzheng Road, Taixing Town, Taixing, 225400, China

**Keywords:** lncRNA GNAS-AS1, lung adenocarcinoma cells, miR-433-3p, Rab3A, apoptosis, EMT

## Abstract

The goal of this study was to demonstrate the functions and specific mechanism of long non-coding RNA (lncRNA) GNAS-AS1 in lung adenocarcinoma. Levels of lncRNA GNAS-AS1, microRNA (miR)-433-3p, and Rab3A were assessed by quantitative real-time PCR (qRT-PCR). The target-binding sites of lncRNA GNAS-AS1, miR-433-3p, and Rab3A were predicted and confirmed by bioinformatics tool (StarBase) and a dual-luciferase reporter system. Cell proliferation and apoptosis were checked using MTT and flow cytometry, respectively. Additionally, the levels of apoptosis-related and epithelial–mesenchymal transition (EMT)-associated genes in A549 cells were analyzed by qRT-PCR and western blot. We found that lncRNA GNAS-AS1 was upregulated, miR-433-3p was low-expressed, and Rab3A was overexpressed in lung adenocarcinoma tissues and cell lines. LncRNA GNAS-AS1 interacted with miR-433-3p and negatively regulated miR-433-3p levels. Rab3A was a direct target of miR-433-3p. Downregulation of lncRNA GNAS-AS1 remarkably suppressed cell proliferation, promoted cell apoptosis, decreased B-cell lymphoma-2 (Bcl-2) expression, enhanced the Bcl-2-Associated X (Bax) level, promoted E-cadherin expression, and reduced N-cadherin and Rab3A levels. However, the miR-433-3p inhibitor reversed all these findings. Similarly, the inhibitory effects of miR-433-3p mimic on A549 cells were reversed by the Rab3A-plasmid. In conclusion, lncRNA GNAS-AS1 downregulation suppressed lung adenocarcinoma cell proliferation and EMT through the miR-433-3p/Rab3A axis.

## 1 Introduction

Lung cancer is the most frequent cancer globally, with a high incidence and mortality rate. Less than 20% of lung cancer patients have a survival period of more than 5 years [1,2]. Invasion and migration are considered the most lethal features of solid tumors, but their molecular mechanism remains unclear [3]. Lung cancer has a poor prognosis related to tumor migration and invasion. Previous studies have confirmed that epithelial–mesenchymal transition (EMT) is vital in promoting tumor metastasis, characterized by the loss of E-cadherin and the increase of vimentin and N-cadherin [4]. Lung adenocarcinoma is the primary lung cancer type, accounting for approximately 40% of lung cancer cases [5]. However, most patients with lung adenocarcinoma develop drug resistance after receiving cytotoxic chemotherapy. Therefore, exploring more effective treatment strategies or new biomarkers for lung adenocarcinoma treatment is of great clinical value.

Long non-coding RNAs (lncRNAs) are a type of RNA transcripts with a length of more than 200 nucleotides [6]. Research has shown that lncRNA is involved in various biological processes, such as cell proliferation, apoptosis, migration, invasion, the cell cycle, and differentiation regulation [7]. LncRNA GNAS-AS1, a newly discovered cancer-associated lncRNA, is abnormally expressed in many tumors, including breast cancer, nasopharyngeal carcinoma, and osteosarcoma. Liu et al. suggested that lncRNA GNAS-AS1 facilitates ER + breast cancer cells progression via the regulation of miR-433-3p/GATA3 axis [8]. Wang et al. indicated that lncRNA GNAS-AS1 promotes nasopharyngeal carcinoma cell migration and invasion by regulating Wnt/β-catenin pathway [9]. Mi et al. revealed the biomarker potential of lncRNA GNAS-AS1 in osteosarcoma prognosis and its effect on cellular function [10]. Moreover, a recent study has reported that lncRNA GNAS-AS1 promoted macrophage M2 polarization and NSCLC cell progression via directly inhibiting miR-4319 expression [11]. However, the functions and molecular mechanism of lncRNA GNAS-AS1 in lung adenocarcinoma remain need further elucidation.

MicroRNAs (miRNAs), non-coding single-stranded RNA molecules, play a significant role in transcriptional gene expression. According to previous studies, miRNAs are closely associated with fundamental cell processes. For instance, Sun et al. found that miR-433-3p suppresses cell growth and enhances chemosensitivity by targeting cAMP response element-binding protein in human glioma [12]. Furthermore, Weng et al. reported that miR-433-3p expression was significantly reduced in NSCLC cells, which inhibited the malignant biological behavior of lung cancer cells [13]. However, its specific role and molecular mechanism in lung adenocarcinoma have not been fully elucidated. Through bioinformatics software analysis, we found that Rab3A is a potential target gene for miR-433-3p. Rab3A, a key regulator for transporting cell products to secretory vesicles and lysosomes, is abnormally upregulated in human insulinoma and hepatocellular carcinoma [14,15]. Nevertheless, the roles of Rab3A in lung adenocarcinoma remain unclear. Therefore, in this study, we decided to explore whether miR-433-3p participates in lung adenocarcinoma by regulating Rab3A expression.

In this study, we hypothesized that lncRNA GNAS-AS1 affects the biological behavior of lung adenocarcinoma cells through the regulation of miR-433-3p/Rab3A axis. Thus, our study was designed to (i) illustrate whether lncRNA GNAS-AS1 was associated with the progression of lung adenocarcinoma, (ii) explain the correlation between lncRNA GNAS-AS1, miR-433-3p, and Rab3A, and (iii) uncover the pathogenesis of miR-433-3p/Rab3A in lung adenocarcinoma occurrence.

## 2 Materials and methods

### 2.1 Collection of clinical samples

Carcinoma tissues and normal paracancer tissues from patients with lung adenocarcinoma (*n* = 30) were collected aseptically at the Taixing People’s Hospital (Taixing, China). The tissues were rapidly frozen and kept in liquid nitrogen. Written informed consent was obtained from each patient before participation. This investigation procedure was authorized by Ethics Committee at Taixing People’s Hospital (Approval number: LSLW2021004).

### 2.2 Cell culture

A549, NCI-H23, and BEAS2B cells were bought from the American Type Culture Collection (ATCC, VA, USA). All cells were seeded in Dulbecco’s modified eagle medium (Gibco; Grand Island, NY, USA) containing 10% FBS (Gibco), 1% penicillin/streptomycin (Gibco), and cultured at 37°C under 5% CO_2_ incubator.

### 2.3 Dual-luciferase reporter assay

Bioinformatics tool (StarBase) was used to predict the potential binding sites between lncRNA GNAS-AS1 and miR-433-3p or miR-433-3p and Rab3A. We conducted WT-Rab3A and MUT-Rab3A 3′-untranslated region luciferase reporter gene plasmids to illustrate the binding sites between Rab3A and miR-433-3p. For dual-luciferase reporter assay, Rab3A wild-type or mutant plasmids combined with miR-433-3p mimic or mimic control were transfected into 293T cells using Lipofectamine 2000 (Invitrogen) following the instructions for 24 h. Luciferase activity was analyzed by the Dual-Luciferase Reporter Assay System (Promega, USA) [16]. The same method was used to confirm the binding site between lncRNA GNAS-AS1 and miR-433-3p.

### 2.4 Quantitative real-time PCR (qRT-PCR) analysis

The total RNA from the lung adenocarcinoma tissues, adjacent normal tissues, A549, NCI-H23, and BEAS2B cells was isolated with the TRIzol reagent (TaKara, Shiga, Japan) based on the manufacturer’s protocol. cDNA was obtained using a PrimeScript RT kit (TaKaRa, China), followed by PCR amplification on ABI PRISM 7500 Fast Real-Time PCR system (Agilent Technologies, USA) with SYBR Green PCR kit (TaKaRa) to examine the levels of lncRNA GNAS-AS1, miR-433-3p, Rab3A, E-cadherin, N-cadherin, Bax, Bcl-2, and GAPDH. Relative quantification was calculated using the 2^−ΔΔCt^ formula [17]. Primer sequences for PCR are listed as follows:

GAPDH forward, 5′-CATCATCCCTGCCTCTACTGG-3′;

reverse, 5′-GTGGGTGTCGCTGTTGAAGTC-3′;

U6 forward, 5′-CTCGCTTCGGCAGCACA-3′;

reverse, 5′-AACGCTTCACGAATTTGCGT-3′;

lncRNA GNAS-AS1 forward, 5′-GACGCCTTTCCTACGG-3′;

reverse, 5′-TGGTAACGCACCTTCG-3′;

miR-433-3p forward, 5′-GCCGAGGAGCCCATCATGAT-3′;

reverse, 5′-CTCAACTGGTGTCGTGGA-3′;

E-cadherin forward, 5′-CGAGAGCTACACGTTCACGG-3′;

reverse, 5′-GGGTGTCGAGGGAAAAATAGG-3′;

N-cadherin forward, 5′-TCAGGCGTCTGTAGAGGCTT-3′;

reverse, 5′-ATGCACATCCTTCGATAAGACTG-3′;

Rab3A forward, 5′-GAGTCCTCGGATCAGAACTTCG-3′;

reverse, 5′-TGTCGTTGCGATAGATGGTCT-3′;

Bcl-2 forward, 5′-GGTGGGGTCATGTGTGTGG-3′;

reverse, 5′-CGGTTCAGGTACTCAGTCATCC-3′;

Bax forward, 5′-CCCGAGAGGTCTTTTTCCGAG-3′;

reverse, 5′-CCAGCCCATGATGGTTCTGAT-3′.

### 2.5 Cell transfection

A549 cells were induced by control-siRNA, GNAS-AS1-siRNA, inhibitor control, miR-433-3p inhibitor, mimic control, miR-433-3p mimic, control-plasmid, and Rab3A-plasmid using Lipofectamine^®^ 3000 reagent (Thermo) for 48 h based on the manufacturer’s protocol. qRT-PCR was conducted to analyze cell transfection efficiency.

### 2.6 MTT assay

To determine cell proliferation, MTT assay was performed [18]. After transfection, A549 cells were implanted into 96-well plates, induced by 10 μL MTT (5 mg/mL), and continuously cultivated for an additional 4 h, the culture medium was dislodged, and 100 µL DMSO was added to dissolve the formazan crystals. Next, the OD_570_ was analyzed by a microplate reader (BioTek, USA) after vibration mixing based on the manufacturer’s instructions.

### 2.7 Flow cytometry analysis

After treatment, A549 cells apoptosis was detected by Annexin-V/propidium iodide Apoptosis Detection Kit (BD Bioscience) at room temperature for 10 min in accordance with the manufacturer’s instructions. Then apoptotic cells were determined by flow cytometer (BD Technologies) and analyzed with Kaluza Analysis (version 2.1.1.20653; Beckman Coulter, Inc.) [19].

### 2.8 Western blot analysis

To determine protein expression, western blot analysis was carried out [20]. Protein extracts from A549 cells were dissolved by RIPA buffer (Solarbio, Beijing) and quantified by BCA Protein Assay Kit (Invitrogen, USA). Each sample was split by sodium dodecyl sulfate-polyacrylamide gel and transferred onto polyvinylidene fluoride membranes. After incubating with 5% non-fat milk in PBST at room temperature for 1 h, the membranes were cultivated in specific primary antibody (cat. no. #14472 for anti-E-cadherin, 135 kDa, 1:1,000, CST, Danvers, MA, USA; cat. no. #4061 for anti-N-cadherin, 140 kDa, 1:1,000, CST, Danvers, MA, USA; cat. no. ab32503 for anti-Bax, 20 kDa, 1:1,000, Abcam, Cambridge, MA, USA; cat. no. ab32124 for anti-Bcl-2, 26 kDa, 1:1,000, Abcam, Cambridge, MA, USA; cat. no. 15029-1-AP for Rab3A, 25 kDa, 1:1,000, Proteintech, Wuhan, China; or cat. no. ab9485 for anti-GAPDH, 37 kDa, 1:1,000, Abcam, Cambridge, MA, USA) overnight at 4°C. Next, membranes were washed and cultivated with a secondary antibody (cat. no. ab7090/6789; 1:2,000; Abcam, Cambridge, MA, USA) for 1 h. The protein signals were visualized by the electrogenerated chemiluminescence method (Cytiva) according to the manufacturer’s protocol.

### 2.9 Statistical analysis

Statistical analysis was analyzed using SPSS11 software. Data are shown as the mean ± standard deviation from three independent experiments. The statistical significance among groups was calculated using a one-way analysis of variance or Student’s *t*-test. *P < 0.05 and **P < 0.01 indicated as statistically significant.

## 3 Results

### 3.1 Expression of lncRNA GNAS-AS1, miR-433-3p, and Rab3A in lung adenocarcinoma tissues and lung adenocarcinoma cells lines

**Figure 1:**
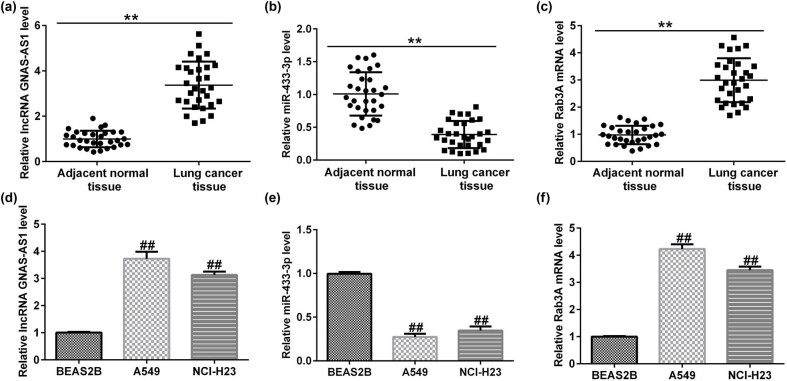
Expression of lncRNA GNAS-AS1, miR-433-3p, and Rab3A in lung adenocarcinoma tissues and lung adenocarcinoma cells. Determination of lncRNA GNAS-AS1 (a), miR-433-3p (b), and Rab3A (c) levels in lung adenocarcinoma tissues and normal paracancer tissues. Detection of lncRNA GNAS-AS1 (d), miR-433-3p (e), and Rab3A (f) expression in lung adenocarcinoma cells lines (A549 and NCI-H23) and BEAS2B cells (as determined by qRT-PCR). **P < 0.01 vs healthy control; ^##^ *P* < 0.01 vs BEAS2B.

To assess the levels of lncRNA GNAS-AS1, miR-433-3p, and Rab3A in 30 lung adenocarcinoma tissues and lung adenocarcinoma cells lines, qRT-PCR was performed. As displayed in Figure 1a–c, lncRNA GNAS-AS1 was upregulated, miR-433-3p was downregulated, and Rab3A was overexpressed in lung adenocarcinoma tissues compared to adjacent noncancerous tissues. Additionally, we observed similar results in lung adenocarcinoma cell lines (A549 and NCI-H23) compared to BEAS2B (Figure 1d–f). Our findings suggested that lncRNA GNAS-AS1 plays a regulatory role in lung adenocarcinoma.

### 3.2 miR-433-3p directly interacted with lncRNA GNAS-AS1

**Figure 2:**
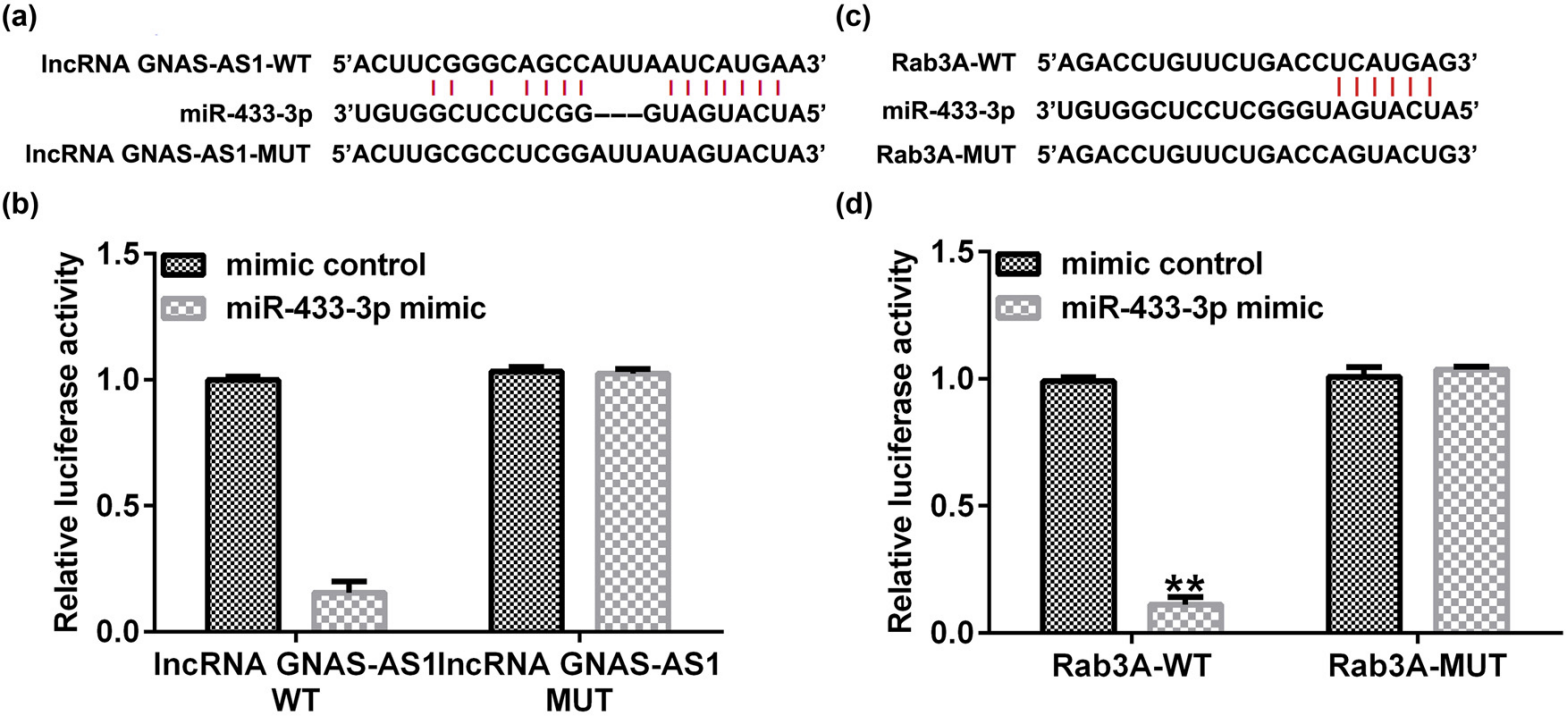
Relationship between lncRNA GNAS-AS1 and miR-433-3p, miR-433-3p and Rab3A. (a) Binding site between lncRNA GNAS-AS1 and miR-433-3p was forecasted by StarBase. (b) Dual-luciferase reporter assay was used to confirm the relationship. (c) Bioinformatics analysis (StarBase) suggested that Rab3A was a latent target of miR-433-3p. (d) Association between miR-433-3p and Rab3A was verified using the dual-luciferase reporter assay. **P < 0.01 vs mimic control.

To illustrate whether lncRNA GNAS-AS1 acts as endogenous competitive RNAs targeting specific miRNAs, a bioinformatics tool (StarBase) was used to predict the candidate site of lncRNA GNAS-AS1. As displayed in Figure 2a, miR-433-3p was a latent target of lncRNA GNAS-AS1. Then, the dual-luciferase reporter gene system confirmed the association between lncRNA GNAS-AS1 and miR-433-3p (Figure 2b). Furthermore, findings in Figure 2c and d revealed that Rab3A directly interacted with miR-433-3p. Our report confirmed that miR-433-3p and Rab3A were associated with lung adenocarcinoma.

### 3.3 LncRNA GNAS-AS1 negatively regulated miR-433-3p expression in A549 cells

**Figure 3:**
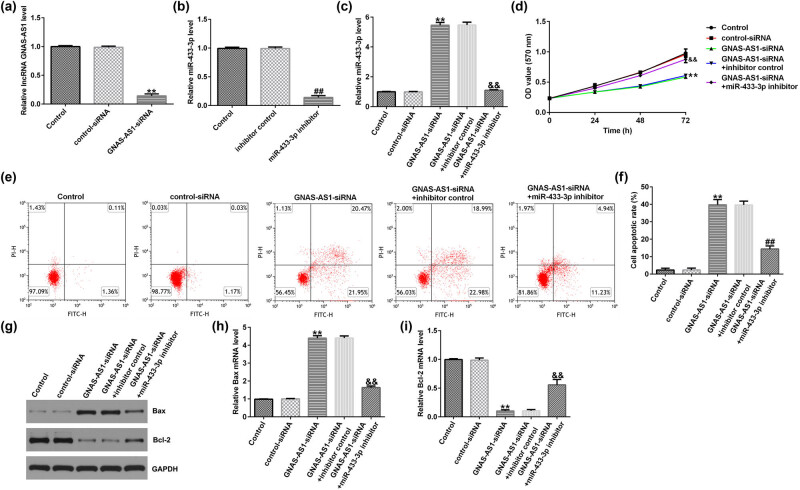
Effects of miR-433-3p inhibitor or GNAS-AS1-siRNA on A549 cell proliferation and apoptosis. Control-siRNA, GNAS-AS1-siRNA, inhibitor control, or miR-433-3p inhibitor were transfected into A549 cells for 48 h. (a) qRT-PCR analysis of lncRNA GNAS-AS1 in control-siRNA or GNAS-AS1-siRNA transfected A549 cells. (b) miR-433-3p expression in inhibitor control or miR-433-3p inhibitor-transfected A549 cells was analyzed using qRT-PCR. (c) qRT-PCR analysis of miR-433-3p in A549 cells. (d) A549 cells viability was measured using the MTT assay. (e) Measurement of apoptotic A549 cells by flow cytometry analysis. (f) Quantitative analysis of apoptotic A549 cells. (g) Western blot analysis of Bax and Bcl-2 expression. (h and i) mRNA levels of Bax and Bcl-2 were evaluated by qRT-PCR. **P < 0.01 vs control-siRNA; ^##^ *P* < 0.01 vs inhibitor control; ^&&^ *P* < 0.01 vs GNAS-AS1-siRNA + inhibitor control.

To evaluate the functions of GNAS-AS1 and miR-433-3p in lung adenocarcinoma cells, control-siRNA, GNAS-AS1-siRNA, inhibitor control, or miR-433-3p inhibitor were transfected into A549 cells for 48 h. The qRT-RCR analysis revealed that GNAS-AS1-siRNA dramatically reduced GNAS-AS1 levels in A549 cells compared to the control-siRNA group (Figure 3a). Moreover, miR-433-3p was markedly downregulated in miR-433-4p inhibitor-transfected cells compared to inhibitor control (Figure 3b). As shown in Figure 3c, GNAS-AS1-siRNA enhanced miR-433-3p level in A549 cells, whereas we observed the opposite results in miR-433-3p inhibitor-transfected cells, demonstrating that GNAS-AS1 negatively regulated miR-433-3p expression in A549 cells.

### 3.4 GNAS-AS1-siRNA suppressed A549 cells proliferation and accelerated cells apoptosis by regulating miR-433-3p

To illustrate the biological functions of A549 cells co-regulated by lncRNA GNAS-AS1 and miR-433-3p, control-siRNA, GNAS-AS1-siRNA, GNAS-AS1-siRNA + inhibitor control, or GNAS-AS1-siRNA + miR-433-3p inhibitor were transfected into A549 cells for 48 h. MTT and flow cytometry analysis demonstrated that GNAS-AS1-siRNA suppressed A549 cell proliferation (Figure 3d) and promoted more apoptotic cells than the control-siRNA group (Figure 3e and f). Furthermore, GNAS-AS1-siRNA increased Bax expression (Figure 3g and h) and reduced Bcl-2 levels in A549 cells (Figure 3g and i) compared to the inhibitor control group. Nevertheless, these observations were eliminated by the miR-433-3p inhibitor, revealing that miR-433-3p downregulation had a proliferation inhibition effect available in A549 cells.

### 3.5 Knockdown of lncRNA GNAS-AS1 suppressed A549 cells EMT

**Figure 4:**
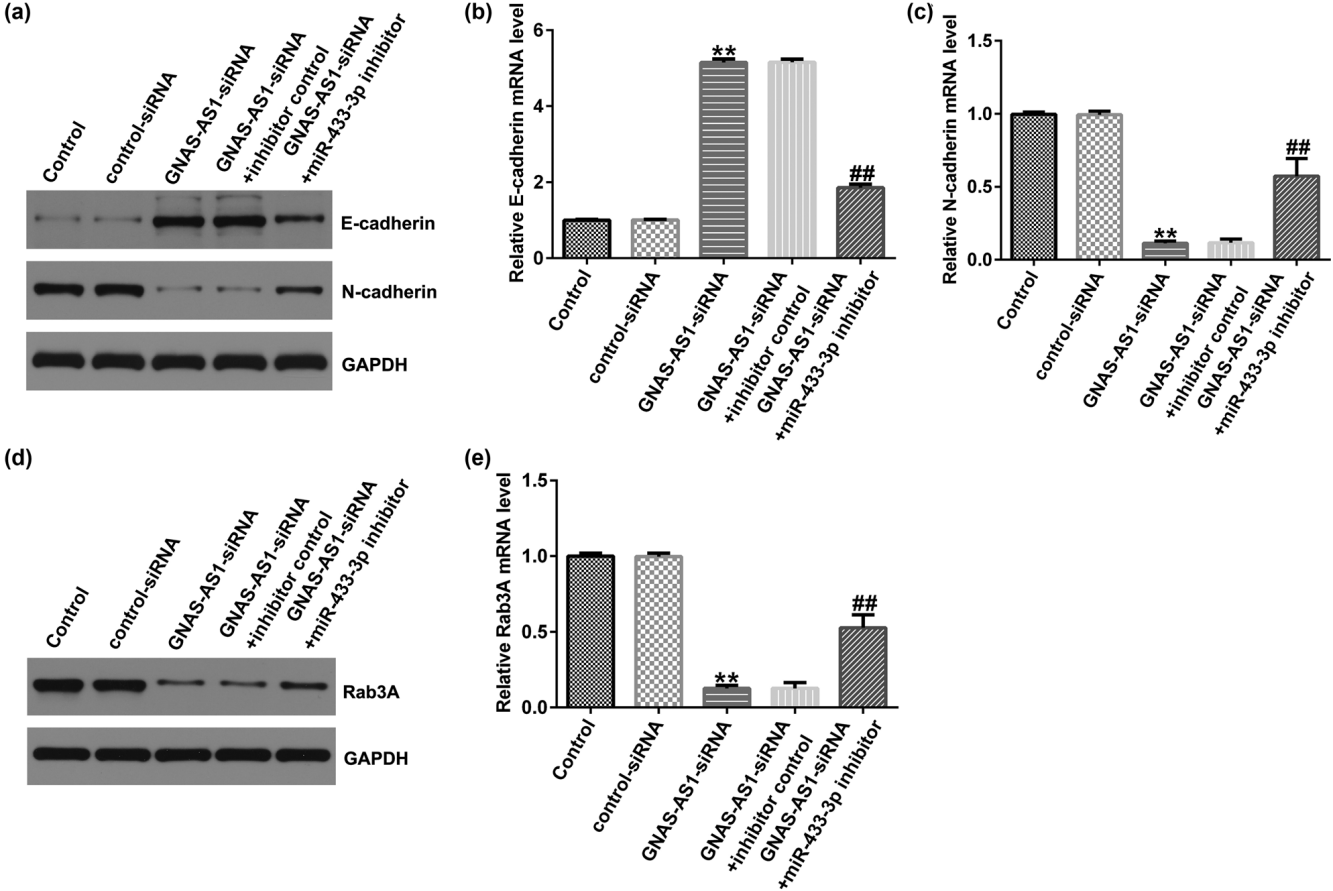
Effects of miR-433-3p inhibitor or GNAS-AS1-siRNA on A549 cells EMT. Control-siRNA, GNAS-AS1-siRNA, GNAS-AS1-siRNA + inhibitor control, or GNAS-AS1-siRNA + miR-433-3p inhibitor were transfected into A549 cells for 48 h. (a) Detection of E-cadherin and N-cadherin expression using western blot. mRNA levels of E-cadherin (b) and N-cadherin (c) in A549 cells were checked by qRT-PCR analysis. (d) Detection of Rab3A expression using western blot. Rab3A (e) mRNA levels in A549 cells were checked by qRT-PCR analysis. **P < 0.01 vs control-siRNA; ^##^ *P* < 0.01 vs GNAS-AS1-siRNA + inhibitor control.

It is well known that EMT is related to the invasion of cancer cells. We further analyzed EMT markers using western blot and qRT-PCR. As displayed in Figure 4a–c, the level of E-cadherin was enhanced, whereas N-cadherin expression was suppressed, when lncRNA GNAS-AS1 was downregulated in A549 cells. In addition, the Rab3A level was signally reduced in the GNAS-AS1-siRNA group compared with the control-siRNA group (Figure 4d and e). However, these findings of GNAS-AS1-siRNA were partially reversed by the miR-433-3p inhibitor. These results demonstrated that GNAS-AS1-siRNA inhibited tumor cell EMT through binding miR-433-3p.

### 3.6 miR-433-3p mimic suppressed cells viability and promoted cells apoptosis in A549 cells

**Figure 5:**
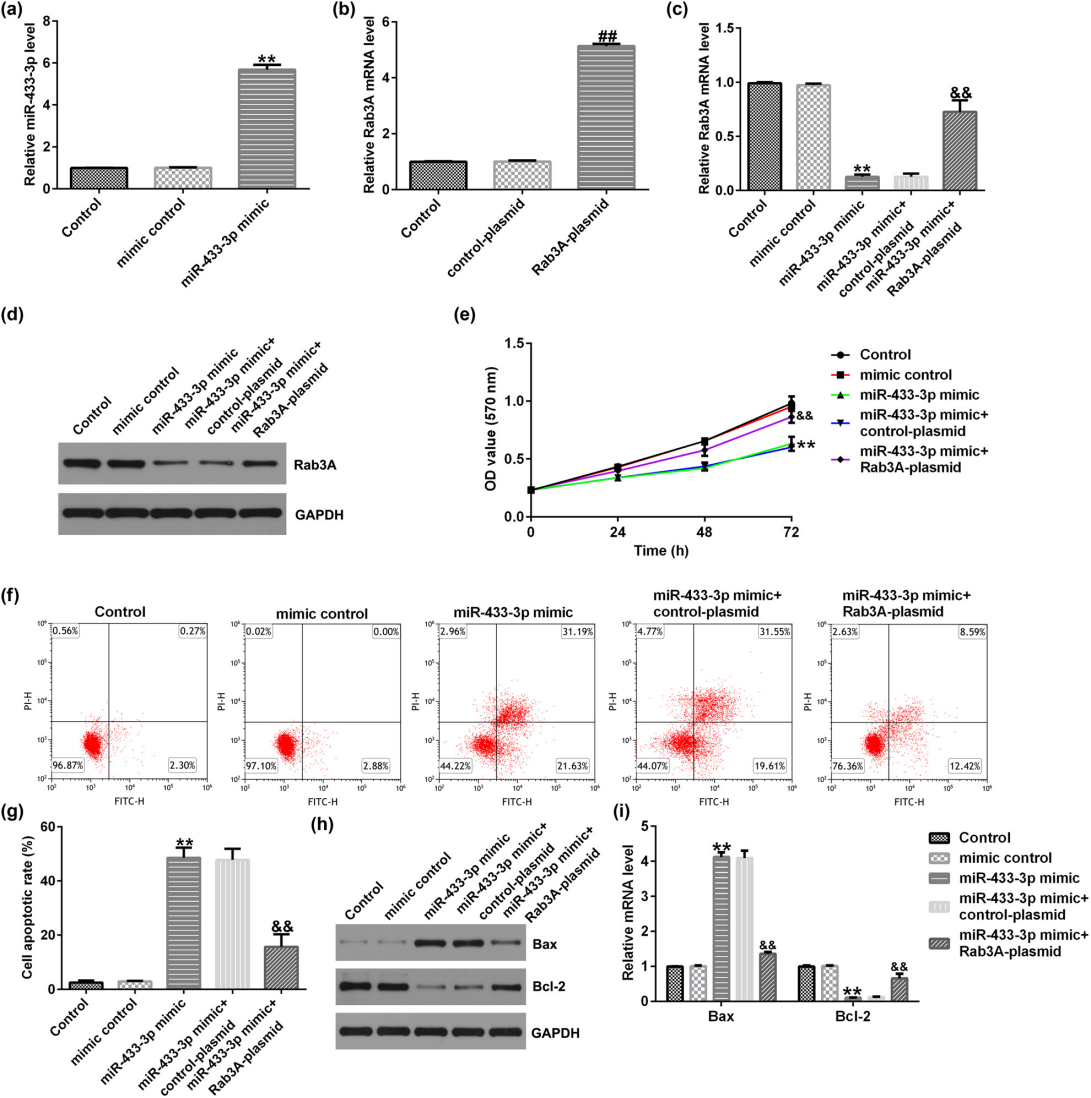
Effects of miR-433-3p mimic or Rab3A-plasmid on A549 cell viability and apoptosis. Mimic control, miR-433-3p mimic, control-plasmid, or Rab3A-plasmid were transfected into A549 cells for 48 h. (a) qRT-PCR analysis of miR-433-3p in mimic control or miR-433-3p mimics transfected A549 cells. (b) Rab3A expression in A549 cells was analyzed using qRT-PCR. (c and d) qRT-PCR analysis and western blot assay of Rab3A in A549 cells. (e) A549 cells viability was checked using the MTT assay. (f) Measurement of apoptotic A549 cells using flow cytometry analysis. (g) Quantitative analysis of apoptotic A549 cells. (h) Western blot analysis of Bax and Bcl-2 expression. (i) mRNA levels of Bax and Bcl-2 expression. **P < 0.01 vs mimic control; ^##^ *P* < 0.01 vs control-plasmid; ^&&^ *P* < 0.01 vs miR-433-3p mimic + control-plasmid.

To illustrate the influence of the miR-433-3p mimic on cell viability and apoptosis, mimic control, miR-433-3p mimic, control-plasmid, and Rab3A-plasmid were transfected into A549 cells for 48 h. As shown in Figure 5a, the miR-433-3p mimic enhanced its miR-433-3p level compared to the mimic control group (Figure 5a). Moreover, Rab3A was upregulated in Rab3A-plasmid transfected A549 cells (Figure 5b). miR-433-3p mimic memorably reduced Rab3A expression in A549 cells, and this inhibition was eliminated in the miR-433-3p mimic + Rab3A-plasmid co-transfected cells (Figure 5c and d), suggesting that miR-433-3p negatively regulated Rab3A expression in A549 cells.

Furthermore, we illustrated the functions of miR-433-3p mimic in A549 cells viability and apoptosis, and the mimic control, miR-433-3p mimic, miR-433-3p mimic + control-plasmid, or miR-433-3p mimic + Rab3A-plasmid was transfected into A549 cells for 48 h. Figure 5e–g indicates that the miR-433-3p mimic reduced A549 cells viability and promoted apoptotic cells. Western blot analysis demonstrated that miR-433-3p mimicked enhanced Bax expression and inhibited Bcl-2 levels (Figure 5h and i) in A549 cells, compared to a mimic control group, whereas the Rab3A-plasmid reversed all these findings. In summary, we found that miR-433-3p was involved in A549 cell proliferation and apoptosis.

### 3.7 Upregulation of miR-433-3p inhibited cell EMT

**Figure 6:**
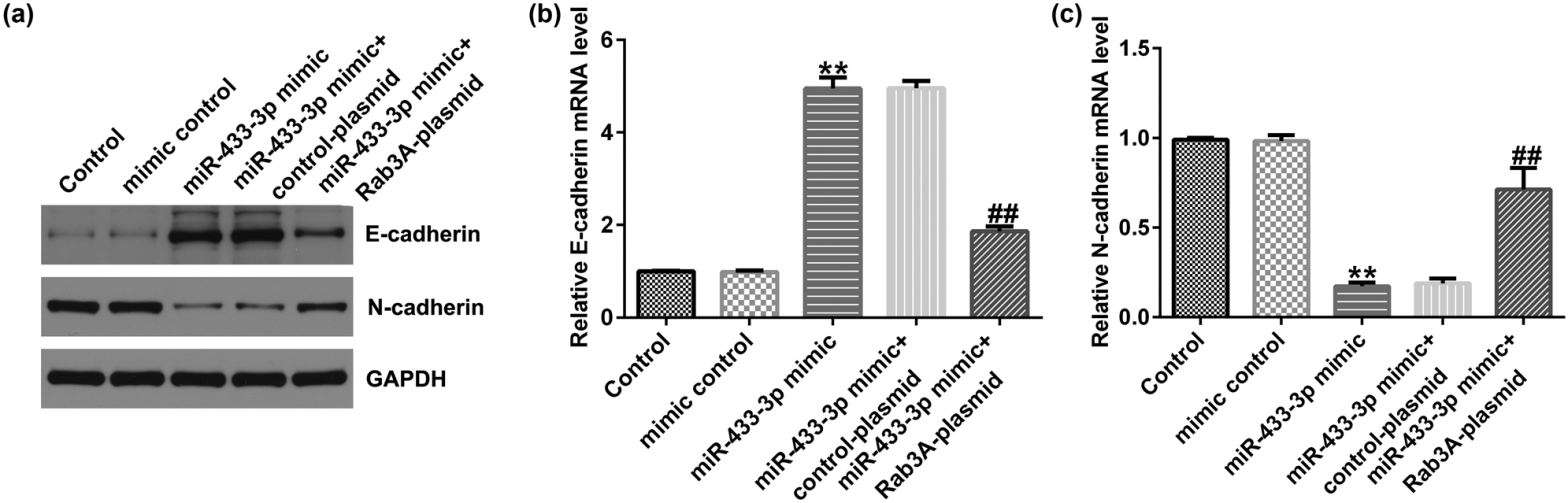
Effects of miR-433-3p mimic or Rab3A-plasmid on A549 cells EMT. Mimic control, miR-433-3p mimic, miR-433-3p mimic + control-plasmid, or miR-433-3p mimic + Rab3A-plasmid were transfected into A549 cells for 48 h. (a) Detection of E-cadherin and N-cadherin expression using western blot assay. mRNA levels of E-cadherin (b) and N-cadherin (c) in A549 cells were checked by qRT-PCR analysis. **P < 0.01 vs mimic control; ^##^ *P* < 0.01 vs miR-433-3p mimic + control-plasmid.

To determine EMT markers in A549 cells after mimic control, miR-433-3p mimic, control-plasmid, or Rab3A-plasmid transfection, western blot and qRT-PCR analysis were used. As presented in Figure 6a–c, the miR-433-3p mimic led to increased E-cadherin expression and reduced N-cadherin level, whereas we observed the opposite findings in the miR-433-3p mimic + Rab3A-plasmid transfected cells. Our results indicated that the miR-433-3p mimicked suppressed tumor cell EMT via targeting Rab3A.

## 4 Discussion

The findings of this study proposed a new modulator mechanism that lncRNA GNAS-AS1 gene silencing inhibits lung adenocarcinoma cell proliferation and EMT and induces cell apoptosis through the miR-433-3p/Rab3A axis, which may provide an effective strategy for lung adenocarcinoma treatment.

Lung cancer has been a primary cause of cancer morbidity and mortality worldwide. Great efforts have been made in lung cancer therapy in the past decades, including surgical resection, chemotherapy, and radiotherapy [21,22]. Lung adenocarcinoma has become the primary lung cancer type because of smoking habits and other lifestyle changes that account for approximately 50% of lung cancer cases worldwide [23]. Unfortunately, most patients with lung adenocarcinoma that received standard cytotoxic chemotherapy eventually developed drug resistance. Hence, focusing on effective strategies or new biomarkers has excellent clinical value for lung adenocarcinoma therapy.

LncRNAs are endogenous molecules with a length of 19–22 nucleotides that play a vital role in multiple tumors, including lung adenocarcinoma [24]. Numerous investigations have evidenced that lncRNAs were associated with biological functions, including cell viability, apoptosis, and metastasis. Moreover, increasing studies have evidenced that lncRNAs function via targeting miRNAs in tumors. For instance, Liang et al. suggested that lncRNA BCRT1 promotes breast cancer progression by targeting the miR-1303/PTBP3 axis [25]. A recent study has reported that lncRNA GNAS-AS1 promoted NSCLC cell progression via regulating miR-4319 [11]. Nevertheless, it is unclear whether lncRNA GNAS-AS1 functions as an essential regulator in lung adenocarcinoma via targeting miR-433-3p. In our study, we concentrated on exploring the mechanism of lncRNA GNAS-AS1 in lung adenocarcinoma.

First, we evaluated lncRNA GNAS-AS1, miR-433-3p, and Rab3A in 30 lung adenocarcinoma tissues, adjacent normal tissues, lung adenocarcinoma cells, and BEAS2B using qRT-PCR analysis. We observed that lncRNA GNAS-AS1 was upregulated, miR-433-3p was low-expressed, and Rab3A was overexpressed in lung adenocarcinoma tissues compared to adjacent noncancerous tissues. These findings were further confirmed in lung adenocarcinoma cell lines through qRT-PCR. Our results agree with other researchers, who have suggested that lncRNA GNAS-AS1 is involved in many cancers [8–11]. Therefore, downregulation of lncRNA GNAS-AS1 may block tumorigenesis in lung adenocarcinoma.

Furthermore, previous investigations have suggested that miRNAs act as oncogenes or inhibitor genes in many cancers [26]. lncRNAs possess vital roles during cancer progression by targeting miRNAs. We further explored the latent targets of lncRNA GNAS-AS1. Our data indicated a latent mechanism of miR-433-3p and Rab3A with lung adenocarcinoma oncogenesis. MiRNA usually functions via interacting with target genes. miR-433-3p, a member of the miR-433 family, exerts multiple functions in human tumorigenesis. You et al. demonstrated that miR-433-3p restrains the proliferation, migration, and invasion of glioma cells via targeting SMC4 [27]. Evidence has revealed that dysregulation of lncRNAs is related to multiple disease progression [28]. We speculated that an altered lncRNA GNAS-AS1 level could change lung adenocarcinoma functions. Our data revealed that lncRNA GNAS-AS1 negatively regulated miR-433-3p expression in A549 cells.

Apoptosis defects are essential for tumor cells, and irritating cell apoptosis may block tumor development [29]. EMT is a key transition stage from epithelial cells to mesenchymal cells, and changes in EMT phenotype and genes can promote tumor invasion and metastasis [30,31]. Therefore, reversing the EMT program of tumor cells would be an effective strategy for cancer therapy [32,33]. During EMT, mesenchymal markers, such as N-cadherin are enhanced, while epithelial markers, such as E-cadherin protein is weakened [34]. We further illustrated whether downregulation of lncRNA GNAS-AS1 affects lung adenocarcinoma cell proliferation, apoptosis, and EMT through miR-433-3p. We found that GNAS-AS1-siRNA led to the suppressing A549 cells proliferation and promoting more apoptotic cells. Bax and Bcl-2 are vital mediators of apoptosis and ultimately contribute to apoptotic cell death [35]. We also determined Bax and Bcl-2 expression and EMT-related expression in A549 cells. Our data indicated that lncRNA GNAS-AS1 knockdown inhibited proliferation and EMT of lung adenocarcinoma cells through binding miR-433-3p. Rescue experiments were conducted to better elucidate the regulatory relationship between miR-433-3p and Rab3A in A549 cells. Then, we explored whether miR-433-3p can affect the proliferation, apoptosis, and EMT of lung adenocarcinoma cells by downregulating Rab3A. We observed that miR-433-3p mimic memorably reduced A549 cells viability, promoted apoptotic cells, enhanced Bax expression, and inhibited Bcl-2 levels in A549 cells, compared with the mimic control group Rab3A-plasmid reversed all these findings. Additionally, the, miR-433-3p mimic led to increased E-cadherin expression and reduced N-cadherin level, whereas we observed the opposite findings in the miR-433-3p mimic + Rab3A-plasmid transfected cells. Our results indicated that the miR-433-3p mimicked suppressed tumor cell EMT via targeting Rab3A.

In summary, our findings identified that downregulation of lncRNA GNAS-AS1 inhibits proliferation and EMT of lung adenocarcinoma cells by regulating the miR-433-3p/Rab3A axis. Our investigations better illustrated the pathogenesis of lung adenocarcinoma and could provide prognostic biomarkers for lung adenocarcinoma treatment. Nevertheless, animal experiments will be more convincing for our conclusions, so our next study will explore the effect of lncRNA GNAS-AS1/miR-433-3p/Rab3A on lung adenocarcinoma *in vivo*.

## 5 Conclusion

This study revealed that inhibition of lncRNA GNAS-AS1 reduced cell proliferation and epithelial–mesenchymal transition, enhanced cell apoptosis through the miR-433-3p/Rab3A axis in lung adenocarcinoma cells, which provides a theoretical basis for lung adenocarcinoma treatment.
